# Immunoregulatory Effects of Stem Cell-Derived Extracellular Vesicles on Immune Cells

**DOI:** 10.3389/fimmu.2020.00013

**Published:** 2020-02-11

**Authors:** Min Xie, Wei Xiong, Zhou She, Zaichi Wen, Amin Sheikh Abdirahman, Wuqing Wan, Chuan Wen

**Affiliations:** ^1^Division of Hematology and Tumor, Children's Medical Center, The Second Xiangya Hospital, Central South University, Changsha, China; ^2^Department of Hepatobiliary Surgery, Sichuan Provincial People's Hospital, Chengdu, China

**Keywords:** extracellular vesicle, microvesicle, exosome, stem/progenitor cell, immune cell, immunoregulatory

## Abstract

Recent investigations on the regulatory action of extracellular vesicles (EVs) on immune cells *in vitro* and *in vivo* have sparked interest on the subject. As commonly known, EVs are subcellular components secreted by a paracellular mechanism and are essentially a group of nanoparticles containing exosomes, microvesicles, and apoptotic bodies. They are double-layer membrane-bound vesicles enriched with proteins, nucleic acids, and other active compounds. EVs are recognized as a novel apparatus for intercellular communication that acts through delivery of signal molecules. EVs are secreted by almost all cell types, including stem/progenitor cells. The EVs derived from stem/progenitor cells are analogous to the parental cells and inhibit or enhance immune response. This review aims to provide its readers a comprehensive overview of the possible mechanisms underlying the immunomodulatory effects exerted by stem/progenitor cell-derived EVs upon natural killer (NK) cells, dendritic cells (DCs), monocytes/macrophages, microglia, T cells, and B cells.

## Introduction

Extracellular vesicles (EVs), now identified as a novel apparatus of intercellular communication, did not garner significant attention previously, although they are currently sought after as a topic of research. EVs have a diameter ranging between 50 and 2,000 nm with a bilayer lipid membrane (1) and comprise parental cell-derived active cargos such as lipids (2), proteins (2, 3), nucleic acids [(DNAs) (4), mRNA (5, 6), microRNAs (miRNAs) (4), non-coding RNAs (7)] and organelles (4, 8). Emerging evidence indicates that double-stranded DNA, DNA-binding histones, and certain miRNAs are not associated with small EVs such as exosomes (9). These inclusions are attached to EV membranes or included within the vesicles (10–12). The components of EVs vary with environmental conditions, cell origin, and cell activation conditions. Moreover, EVs demonstrate significant age-dependent differences in their pro-inflammatory miRNA profile (12). To date, most cell types [including stem cells (SCs)/progenitor cells] have been shown to release specific EVs (13–15), and a growing body of evidence indicates that EVs derived from stem/progenitor cells contribute to immunomodulation responses (16). EVs are detected in all body fluids and serve as a basis for liquid biopsy (17). This review focuses on the mechanisms underlying the immunoregulatory effects exerted by stem/progenitor cell-derived EVs on natural killer (NK) cells, dendritic cells (DCs), monocytes/macrophages, microglia, T cells, and B cells. Defining the mechanism of action of SC-derived EVs (SC-EVs) will facilitate development of novel therapeutic approaches on the basis of the synergistic effects of EVs with other beneficial molecules or drugs with complementary effects.

## Biological Properties of Extracellular Vesicles

An increasing number of studies report the process of secretion of EVs by various cells (15, 18). A large number of protuberances and pits on the membrane surface of viable SCs are observed to be dynamic; the protuberances may shed as microvesicles (MVs), and the pits may form when multivesicular bodies (MVBs) fuse with the plasma membrane to release exosomes (18).

EVs are classified into three main categories on the basis of their size and biogenesis: (1) Exosomes (50–120 nm) are produced through the inward invagination of the endosomal membrane. First, the inward budding of the plasma membrane leads to formation of the early endosome, followed by formation of intraluminal vesicles (ILVs) by inward budding of the limiting membrane inside MVBs, followed by release of ILVs from MVBs to the extracellular space after fusing with the plasma membrane, leading to formation of exosomes (1). Biogenesis within endosomes (now known as MVBs) is distinctive to exosomes. (2) MVs (200–2,000 nm) develop as membrane protrusions and eventually form bulges that detach directly (17). (3) Apoptotic bodies (Abs) (500–2,000 nm) characterized by the presence of organelles within the membrane enclosed vesicles released by cells undergoing apoptosis (19). Furthermore, a growing body of evidence suggests that apoptotic cell-derived EVs (ApoEVs) play a significant role in immunomodulation. For example, ApoEVs promote phagocyte recruitment to clear apoptotic cells, present antigen to T cells, facilitate immune response of DCs, and induce infection (20, 21). Thus, SC-EVs may be widely used in treatment of autoimmunity, cancer, and infection in the future. Limited information is available about the functional significance of ApoEVs, apart from its role in fragmentation of cells undergoing apoptosis and the immunomodulatory activities of other cell-derived ApoEVs (21). Therefore, we focus on the immunomodulatory effects of SC exosomes and SC-MVs in our review.

Owing to the overlap in size and density, the term “EVs” usually refers to exosomes and MVs. Intercellular communication is achieved through a variety of pathways, such as cell–cell contact (22), tunnel nanotubes (23), and paracrine mechanisms. Research has indicated that EVs transfer signaling molecules from one cell to another cell or into various body fluids through a paracrine mechanism, thus regulating the gene expression and phenotypic transformation of target cells through a continuous secretion-uptake process. Therefore, EVs are significant as information vehicles. EVs are taken up by target cells through direct membrane fusion, receptor-mediated phagocytosis, and several other internalized mechanisms (24), leading to subsequent activation of signal transduction pathways (14, 25) and involvement in various physiological and pathological processes *in vivo*, such as immune response and cell phenotypic transformation.

## Bidirectional Interaction of Stem Cells With Immune Cells Through Their Respective Extracellular Vesicles

**Figure 1:**
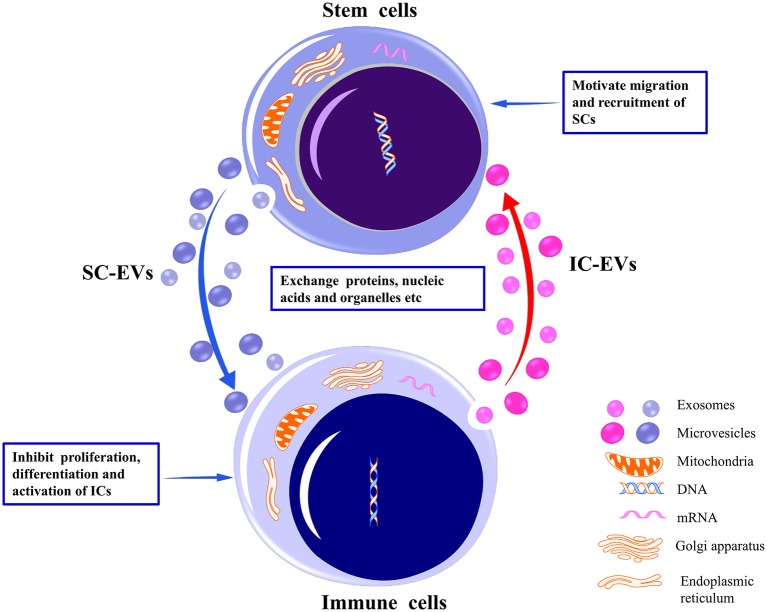
Bidirectional interaction between stem cells and immune cells through their respective EVs. By delivering proteins, nucleic acids, organelles, etc., EVs released from stem cells may inhibit the proliferation, differentiation, and activation of the immune cells to induce immunotolerance. Conversely, EVs released from immune cells may motivate the migration and recruitment of stem cells to promote tissue repair. EVs, extracellular vesicles; SCs, stem cells; ICs, immune cells.

SCs are capable of self-renewal and indefinite proliferation, participating in maintenance of cell cycle, tissue repair and regeneration, and immune response regulation. SC-EVs are internalized by target cells primarily through specific receptor–ligand interaction modes to exert biological functions (26–28). Intercellular communication between SCs and immune cells is achieved through their respective EVs. For example, SC-EVs internalized by immune cells inhibit the proliferation and activation of the latter (29, 30) (Figure 1). SC-EVs preferentially accumulate in injury sites to inhibit the pro-inflammatory response of immune cells (31). EVs derived from immune cells are also internalized by SCs to promote recruitment and migration of SCs (32) (Figure 1). Additionally, immune cells and tumoral SCs have been observed to restrict each other through EVs. For example, activated CD8+ T cell-derived EVs were observed to prevent tumor progression by EV-mediated depletion of mesenchymal stromal/stem cells (MSCs) associated with tumor expansion in tumor environment (33). Glioblastoma SCs (GSCs)-derived EVs induced inclination of human monocytes toward the immunosuppressive M2 phenotype expressing programmed death ligand-1 (PD-L1), leading to the spread of tumor cells (34). Overall, the bidirectional interaction of EVs secreted by SCs and by immune cells has provided a theoretical basis for exploring tissue/organ repair and antitumor mechanisms. In this review, we focus on the regulatory potential of SC-EVs on immune cells.

## Immunomodulatory Potential of Stem Cell-Derived Extracellular Vesicles on Immune Cells

### Natural Killer Cells

SC-EVs primarily exert immunosuppressive effects on NK cells, including recruitment, proliferation, activation, and release of cytotoxic substances. For example, human umbilical cord MSC-derived EVs (hUC-MSC-EVs) demonstrated a protective role in rats with renal ischemia–reperfusion injury through downregulation of the renal expression of C-X3-C motif chemokine ligand-1 (CX3CL1) and toll-like receptor-2 (TLR-2), and transfer of various miRNAs, thus inhibiting the CD3-CD161+NK infiltration (35) (Table 1). In an experiment on human graft-vs.-host disease (GVHD), MSC exosomes were shown to reduce the release of interferon gamma (IFN-γ) and tumor necrosis factor alpha (TNF-α) by activated NK cells, alleviating the inflammatory response (100). In addition, the anti-inflammatory molecules contained in MSC exosomes, such as interleukin 10 (IL-10), transforming growth factor-β1 (TGF-β1), and human leukocyte antigen-G (HLA-G), are also believed to exert immunoprotective effects (100). Human fetal liver MSC-EVs have been reported to inhibit the proliferation and activation of CD56-dim/CD56-bright NK cells and to suppress the cytotoxic degranulation capacity of NK cells on target cells *in vitro* (95). A possible mechanism by which MSC-EVs exert these immunomodulatory effects on NK cells could be through the TGF-β expression on their membranes mediating downstream TGF/Smad2/3 signaling (95) (Table 1). These findings suggest that SC-EVs play a therapeutic role in suppressing the lethality of NK cells, which serves as a theoretical basis for disease treatment or drug development.

**Table 1:** Immunoregulatory potential and mechanism of SC-EVs on immune cells.

**SC type releasing EVs**	**Models**	**Transferring materials**	**Target cells**	**Molecular mechanisms**	**Biological effects**	**References**
hUC-MSCs	Renal IRI rat model	miRNAs	Injured kidney	Downregulate TLR-2 and CX3CL1	Promote NK cell suppression and ameliorate renal ischemia–reperfusion injury	(35)
hUC-MSCs	Severe burn rat model	miR-181c	Macrophages	Inhibit NF-κB/p-P65 signal pathway	Reduce macrophage activation and alleviate burn-induced inflammation	(36)
hUC-MSCs	ALF mice model	—	Macrophages	Inhibit TXNIP/NLRP3 inflammasome	Reduce macrophage activation and improve liver function	(37)
hUC-MSCs	AAA mice model	miR-147	Macrophages	—	Induce M1 suppression in aortic smooth muscle cells and mitigate AAA formation	(38)
hUC-MSCs (LPS-pretreated)	Cutaneous wound of diabetic rat model	Let-7b	Macrophages	Suppress TLR4/NF-κB/STAT3/AKT pathway	Promote M2 induction and diabetic cutaneous wound healing	(39)
hUC-MSCs (IL-1β pretreatment)	Sepsis mice model	miR-146a	Macrophages	Target the IRAK1, TRAF6, and IRF5 signaling cascades	Promote M2 induction and prolong the survival of mice with sepsis	(40)
hUC-MSCs	Retinal laser injury mice model, EAU rat model	—	The retina cells	Downregulate MCP-1	Inhibit macrophage infiltration and protect the retina from inflammatory injury	(41, 42)
hUC-MSCs	*In vitro*	CD73 expressing	T cells	—	Suppress T cell proliferation and induce immunosuppressive response	(11)
hUC-MSCs (HLA light chain B2M deletion)	Myocardial infarction rat model	miR-24	Cardiomyocytes	B2M-UCMSC-exosomes/miR-24/Bcl-2-like protein 11(Bim) pathway	Inhibit CD8+ immune rejection and cardiomyocytes apoptosis	(43)
hUC-MSCs	GVHD mice model	—	CD8+ T, Th cells	—	Suppress CD8+ T cells, switch the immune response from Th1 cells to Th2, prevent life-threatening GVHD after allo-HSCT	(44)
hUC-MSCs	Contact hypersensitivity mouse model	—	Tc1 cells, Th1 cells, Tregs	Target STAT1	Suppress Tc1 and Th1 cells, induce Tregs, and exert therapeutic effect	(45)
hUC-MSCs	EAU rat model	—	The retina cells	Downregulate expression of CCL21	Reduce T cell infiltration and protect the retina from inflammatory injury	(42)
hUC-MSCs	Perinatal brain injury rat model	—	Microglia	Suppress TLR4/CD14 signaling pathway (NF-κB/MAPK family members ERK1/2, p38, and JNK)	Prevent and treat perinatal brain injury	(46)
hAD-MSCs (hypoxic pretreated)	Skeletal muscle injury mice model	—	Injured muscle cells	Upregulate CCL2	Increase M2 macrophage infiltration and promote M2 induction and injury site recovery	(47)
hAD-MSCs (IFN-γ stimulated)	*In vitro*	—	CD14+CD16+ Monocytes	—	Induce apoptosis of the targets cells	(48)
hAD-MSCs	Experimental allergic asthma mice model	—	T cells	—	Switch the immune response from Th2 cells to Th1 and reduce inflammation and tissue remodeling	(49)
mAD-MSCs	ALF mice model	miR-17	Macrophages	Inhibit TXNIP/NLRP3 inflammasome	Reduce macrophage activation and improve liver function	(50)
mAD-MSCs	T1D mice model	—	T cells	—	Regulate the immune response axis of Th17/Tregs and prevent T1D progressing	(51)
mAD-MSC	*In vitro*	—	Macrophages, DCs, Th2 cells	—	Promote M2 polarization and DC maturation to ameliorate Th2-mediated inflammation response	(52)
mAD-SCs	Diet-induced obesity mice model	Phosphorylated STAT3	Macrophages	—	M2 induction in WAT and improve systemic metabolic homeostasis	(53)
mAD-SCs	EAE mice model	—	T cells	Inhibit integrin-dependent chemokine pathway	Suppress activated T cell adhesion and ameliorate chronic inflammation	(54)
rAD-MSC	Myocardial infarction rat model	—	Macrophages	Activate S1P/SK1/S1PR1 signaling pathway	M2 induction and meliorate cardiac damage	(55)
rAD-MSCs	HCC rat model	β-Catenin	NK T cells	—	Promote NK-T cell survival and migration, increase NK-T cell antitumor	(56)
rAD-SCs	*In vitro*	—	Microglia	Inhibit NF-κB/MAPK family member signaling pathway	Decrease cytotoxicity of activated microglia	(57)
rAD-SCs miRNA-126-modified	Stroke rat model	miRNA-126	Microglia	—	Treatment for stroke	(58)
rAD-SCs (overexpressed miR-30d-5p)	Acute ischemic stroke rats model	miR-30d-5p	Microglia	Suppress the expression of 3′UTR of both Beclin-1 and Atg5	Inhibit microglial polarization to M1 and decrease the cerebral injury area of infarction	(59)
mBM-MSCs	Allogeneic kidney graft mice model	Micro-146a	DCs	—	Inhibit DC maturation, promote allogeneic kidney graft survival	(60)
rBM-MSCs (IDO1 overexpressing)	Cardiac allografts rat model	FHL-1 protein, miR-540-3p	DCs, T cells	—	Induce DC immaturity, indirectly regulate T cell immune response, promote immunotolerance of cardiac allografts	(61)
hBM-MSCs	*In vitro*	miR-21-5p	DCs, T cells	—	Attenuate DC maturation and function as well as inflammatory response of T cells	(62)
mBM-MSCs	ApoE^−/−^ atherosclerosis mice model	miR-let7	Macrophages	Suppress IGF2BP1/PTEN pathway in the plaque	Reduce macrophage infiltration to meliorate atherosclerosis	(63)
mBM-MSCs	Cardiomyocyte injury in polymicrobial sepsis mice model	miR-223	Macrophages, cardiomyocyte	Downregulate expression of Stat3 and Sema3A proteins	Attenuate inflammatory response and exert cardioprotection	(64)
rBM-MSCs	*In vitro*	—	Macrophages	Target AKT1/AKT2 signaling pathway and suppress the NF-κB signaling pathway	M2 induction and alleviate inflammation	(65)
hBM-MSCs	ARDS mice model	Functional mitochondria	Macrophages	Enhance macrophage oxidative phosphorylation	M2 induction and ameliorate lung injury	(8)
mBM-MSCs	*In vitro*	—	Macrophages	Downregulate expression of CCR7	Promote M2 induction and guide immunotolerance	(27)
mBM-MSCs	ApoE^−/−^ atherosclerosis mice model	miR-let7	Macrophages	Inhibit HMGA2/NF-κB signal pathway	M2 induction and ameliorate atherosclerosis	(63)
hBM-MSCs (Hypoxia prechallenged)	Non-small cell lung cancer cell xenograft mice model	miR-21-5p	Macrophages	Downregulate expression of PTEN gene and promote p-Akt/p-STAT3 signal pathway	M2 induction and promote non-small-cell lung cancer cells growth and mobility	(66)
mBM-MSCs	Dilated cardiomyopathy mice model	—	Macrophages	Activate JAK2-STAT6 signal pathway	Promote M2 induction and ameliorate myocardial inflammation	(67)
mBM-MSCs	Myocardial IRI mouse model	miR-182	Macrophages	Target TLR4/NF-κB/PI3K/Akt pathway	Promote M2 induction and attenuate myocardial IRI	(68)
mBM-MSC	Ulcerative colitis mice model	—	Macrophages	Target JAK1/STAT1/STAT6 signaling pathway	M2 induction and exert therapeutic effects	(69)
hBM-MSCs	Skin wound-healing mice model	miR-223	Macrophages	Target pknox1	M2 induction and accelerate wound healing	(70)
mBM-MSCs	IRI renal injury mice model	CCR2 proteins	Free CCL2	Inhibit NF-κB/p-P65 signaling pathway	M1 suppression and promote the recovery of kidney injury	(26)
hBM-MSCs	aGVHD murine model	miR-125a-3p	T cells	—	Preserve the circulative naive T cells and prolong the survival	(71)
hBM-MSC	Cerebral apoplexy rat model and stroke mice model	—	T cells, B cells, NK cells	—	Attenuate T cell, B cell, and NK cell lymphopenia and prevent postischemic immunosuppression	(72, 73)
mBM-MSCs	Inflammatory arthritis mice model	—	T cells	—	Switch the immune response from Th1 cells to Th2 to therapy the arthritis	(29)
hBM-MSCs	*In vitro*	—	T cells	—	Induce conversion of Th1 into Th2 cells	(74)
hBM-MSCs	Severe refractory asthma mice model	—	T cells	—	Switch the immune response from Th2/Th17 cells to Th1 and ameliorate airway inflammation	(75)
hBM-MSCs	*In vitro*	—	T cells	—	Regulate the immune response axis of Th17/Tregs	(74)
hBM-MSCs	T1D patient	PGE2, TGF-β	T cells	—	Regulate the immune response axis of Th17/Tregs and prevent T1D progressing	(76)
hBM-MSCs	T1D patient	—	DCs, T cells	—	Induce DC immaturity, inhibit differentiation of Th1 and Th17 cells, increase Tregs to induce immunotolerance	(77)
mBM-MSCs	Tight-skin mice model	miR-151-5p	The recipient BM-MSCs, Th2 cells	Suppress IL4Rα/mTOR pathway	Inhibit Th2 cell immune response to therapy systemic sclerosis	(78)
hBM-MSCs	Human-into mouse xenogeneic GVHD model	Adenosine signaling	Th1 cells	—	Induce the apoptosis of Th1 cells and promote immune suppression	(79)
hBM-MSCs	*In vitro*	—	B cells	Affect mRNA expression of B cells	Inhibit the proliferation and function of B-lymphocytes	(80)
hBM-MSC (IFN-γ and TNF-α pretreated)	*In vitro*	miR-155-5p	B cells	Downregulation of PI3K-AKT signaling pathway and modulation of the reorganization of actin cytoskeleton	Inhibit the proliferation and activation of B cells	(81)
hBM-MSCs	*In vitro*	—	CLL B cells	Induce gene expression profile modifications	Promote the CLL progress	(82)
mBM-MSCs (Irradiated)	Inflammatory arthritis mice model, osteoarthritis mice model	—	B cells, plasma cells	—	Repress the activation of B cells, inhibit plasma cell differentiation, and induce IL-10-expressing Breg cells and exert therapeutic effects	(29, 83)
hpBM-MSCs	*Ex vivo In vitro*	Proteins	Plasma cells	—	Promote the differentiation and maturation programs from early circulating antibody-secreting cells to long-lived plasma cells	(3, 84)
mBM-MSCs	*In vitro*	—	Microglia	Suppress phosphorylation of ERK1/2, JNK, and p38 molecules	Inhibit the activation of microglia	(85)
B-MSCs	Traumatic spinal cord injury rat model	—	Microglia, astrocytes	Suppress A1 neurotoxic reactive astrocytes induced by activated microglia	Repair traumatic spinal cord injury	(86)
hAF-SCs	Osteoarthritis rat model	TGF-β	Macrophages	—	M2 induction and promote cartilage repair	(87)
hAF-SCs (IFN-γ treated)	Allograft mice model	IDO1 proteins	T cells	—	Decrease T cell proliferation, increase Tregs, and promote allograft survival	(88)
mESCs	Cardiomyopathy mice model	—	Macrophages	Suppress phosphorylation of MyD88, P38, and JNK molecules	M2 induction and reduce doxorubicin-induced pyroptosis and cardiac remodeling	(89)
mESCs	Implanted lung adenocarcinoma mice model	GM-CSF-expressing	CD8+ T cells, Tregs	—	Increase CD8+ T cells, inhibit Tregs in tumor, activate CD8+ effector cells within the tumors, prophylactic vaccine for cancer prevention	(90)
hESC-MSCs	Allogeneic skin graft mice model	TLL4	Monocytes, T cells	—	M2 induction and mediate differentiation of CD4+ T cells to Treg and enhance the survival of allogeneic skin	(91)
hWJ-MSCs	Ischemic AKI rat model	miRNAs	Endothelial cells of glomerulus and vessels	Downregulate expression of CX3CL1	Reduce macrophages infiltration and renal injury	(28)
cWJ-MSCs	*In vitro*	TGF-β, adenosine signaling	T cells	—	Inhibit CD4 +T cells proliferation	(92)
hPDL-SCs (LPS-pretreated)	*In vitro*	DNA	Macrophages	—	M1 induction	(93)
hPDL-SCs (LPS-stimulated)	Chronic periodontitis	miR-155-5p	T cells	Target sirtuin-1	Regulate the immune response axis of Th17/Tregs and reduce the further deterioration of periodontitis	(94)
hFL-MSCs	*In vitro*	TGF-β	NK cells	Inhibit the nuclear translocation of phosphorylated Smad2/3 in TGF/Smad pathway	Impair NK cells function	(95)
rCD105(+) renal CSCs	*In vitro*	HLA-G	DCs, T cells	—	Inhibit DC maturity, indirectly regulate T cell immune response, promote cancer progression	(96)
h-end-MSCs	*In vitro*	TGF-β	T cells	—	Suppress CD4+ T cell activation	(10)
hNSCs	Thromboembolic stroke murine model	—	Macrophages, T cells	—	Regulate the immune response axis of Th17/Tregs and exert therapeutic effects and improve prognosis	(31)
hMSCs (protein-free medium activated)	T1D and uveoretinitis murine model	—	DCs, T cells	—	Induce DC immaturity and inhibit Th1 and Th17 cells to balance immune responses	(97)
hGSCs	*In vitro*	—	CD14+ monocytes, T cells	—	Involved in the conversion of monocyte phenotypes and in inhibition of T cell immune response	(98)
iPSC-MSCs (protein-free medium activated)	Sjögren's syndrome mouse model	—	APCs, T cells	—	Inhibit Tfh and Th17 cells and prevent SS progression	(99)

*SCs, stem cells; EVs, extracellular vesicles; SC-EVs, stem cell-derived extracellular vesicles; hUC-MSCs, human umbilical cord mesenchymal stem cells; hAD-MSCs, human adipose mesenchymal stem cells; mAD-SCs, murine adipose stem cells; mAD-SCs, murine adipose stem cells; hpBM-MSCs, human primary bone marrow mesenchymal stem cells; mBM-MSCs, murine bone marrow mesenchymal stem cells; hAF-SC, human amniotic fluid stem cells; hESC-MSCs, embryonic stem cell-mesenchymal stem cells; cWJ-MSCs, canine Wharton's jelly mesenchymal stem cells; hPDL-SCs, human periodontal ligament stem cells; hFL-MSCs, human fetal liver mesenchymal stem cells; rCD105(+) renal CSCs, rat CD105+ renal cancer stem cells; h-endMSCs, human endometrial mesenchymal stem cells; hNSCs, human neural stem cells; hGSCs, glioma stem cells; iPSC-MSCs, induced pluripotent stem cell-mesenchymal stem cells; IDO1, indoleamine-2,3-dioxygenase-1; LPS, lipopolysaccharide; IRI, ischemia–reperfusion injury; ALF, acute liver failure; AAA, abdominal aortic aneurysm; ARDS, acute respiratory syndrome; AKI, acute kidney injury; EAU, experimental autoimmune uveitis; aGVHD, acute graft-vs.-host disease; HCC, hepatocellular carcinoma; T1D, type 1 diabetes; EAE, experimental autoimmune encephalomyelitis; HLA-G, human leukocyte antigen-G; CCR2, C-C motif chemokine receptor-2; TLL-4, toll-like ligand 4; NKs, natural killer cells; DCs, dendritic cells; APCs, antigen presenting cells; CLL B, chronic lymphocytic leukemia B cells; CX3CL-1, C-X3-C motif chemokine ligand-1; CCL-2, C-C motif chemokine ligand-2; MCP-1, monocyte chemotactic protein-1; CCR-7, C-C motif chemokine receptor-7; WAT, white adipose tissue; TLR-2, toll-like receptor-2; CX3CL-1, C-X3-C motif chemokine ligand-1; CCL-21, C-C motif chemokine ligand-21; allo-HSCT, allogeneic hematopoietic stem cell transplantation; MAPK, mitogen-activated protein kinase; PGE2, prostaglandin E2; GM-CSF, granulocyte-macrophage colony-stimulating factor*.

### Dendritic Cells

DCs are classic antigen-presenting cells (APCs) with a significant role in adaptive immune response. DCs internalize and process antigens, followed by upregulation of the expression of class II major histocompatibility complex (MHC II) and T cell costimulatory molecules (CD80 and CD86) on their surfaces. The processed antigens are then docked onto MHC II molecules, leading to their transformation into APCs and conversion from immature DCs (iDCs) to mature DCs (mDCs) (101).

SC-EVs have been observed to exert immunosuppressive effects on DCs primarily through inhibition of DC maturation and activation, which hardly affects the proliferation and apoptosis of DCs (60–62, 77, 96, 97). For example, SC-EVs were observed to indirectly inhibit the immune response of T cells by inducing production of immature IL-10-secreting DCs through downregulation of MHC class II and/or costimulatory molecule expression on the surface of DCs (77, 97). One possible mechanism of action mediated by SC-EVs is to upregulate micro-146a expression, downregulate FAS gene expression in DCs, and induce production of an immature phenotype of DCs, followed by inhibition of IL-12 production (60) (Table 1). Another possible mechanism might be related to the immunosuppressive effect exerted by SC-EVs enabled by upregulation of anti-inflammatory HLA-G molecule expression on SC-EVs (96) (Table 1). In addition, the biological effect of EVs reportedly depends on the engineered SCs. For instance, exosomes secreted by indoleamine-2,3-dioxygenase-1 (IDO1)-overexpressing rat bone marrow MSCs (BM-MSCs) increased the expression of both miR-540-3p and immunoregulatory protein FHL-1 and induced production of a low-activity phenotype of DCs, thus inhibiting the proliferation of T cells (61) (Table 1). In other words, SC-EVs suppressed the ability of APCs to create an immunotolerant environment that is advantageous for graft survival (60) and tumor cell escape (96). Investigation of the beneficial or harmful effects of SC-EVs facilitates the understanding of biological mechanisms of diseases and possible methods for controlling them.

### Macrophages/Monocytes

#### Effects of Stem Cell-Derived Extracellular Vesicles on Macrophage Polarization and Homeostasis

SC-EVs have been shown to polarize macrophages to the alternate phenotype. On the one hand, SC-EVs directly or indirectly inhibit the inflammatory reaction of macrophages. For example, (1) SC-EVs directly act on pro-inflammatory macrophages by inhibiting their infiltration (63) (Table 1) and activation (36–38, 50, 64) (Table 1) and by regulating their phenotype polarization from pro-inflammatory M1 toward anti-inflammatory M2, facilitating low expression of pro-inflammatory molecules IFN-γ and TNF-α; contrarily, they enhance the expression of anti-inflammatory molecule IL-10 to induce immunotolerance (6, 8, 27, 39, 40, 53, 55, 63, 65–70, 87, 89) (Table 1). (2) SC-EVs express chemokine receptors (26) (Table 1) and indirectly promote the infiltration of anti-inflammatory M2 macrophages (47) or prevent the migration of pro-inflammatory M1 macrophages through interaction with chemokine ligands expressed on other tissues and cells (28, 41, 42, 47) (Table 1). In addition, as reported by a study, SC-EVs downregulate the production of IL-23 and IL-22 and upregulate anti-inflammatory prostaglandin E2 (PGE2) by indirectly repressing the function of T helper type 17 (Th17) cell or by inducing conversion of Th17 cells into regulatory T cells (Tregs) (102). As a result, SC-EVs induced conversion of activated regulatory macrophages (Mregs) from a pro-inflammatory phenotype to an *alternative* anti-inflammatory phenotype and eventually promoted the reduction of severe inflammation (102). On the other hand, SC-EVs also promote inflammatory reactions of macrophages. For example, the DNA in the outer membrane of EVs derived from lipopolysaccharide (LPS)-preconditioned periodontal ligament SCs (PDL-SCs) synergized with peripheral environmental IFN-γ to promote M1 polarization of macrophages and expression of high levels of pro-inflammatory molecules IL-6 and TNF-α, resulting in teeth damage (93) (Table 1). This finding suggests that the EV-bound DNA might be a potential therapeutic target for periodontitis. A study on a mice model with silicosis that focused on the double-edged effect of SC-EVs on macrophages using different cargos within EVs revealed notable details. The study showed that MSC transferred mitochondria and miRNAs to human macrophages using MSC-MVs and MSC exosomes, respectively (4). MSCs donated their mitochondria to macrophages to enhance the bioenergetics of macrophages though MV-mediated transfer under oxidative stress. However, MSC-exosome-transferred miRNAs were responsible for targeting MYD88-dependent inflammatory centers to suppress TLR/NF-κB signaling pathway and macrophage activation (4). The dual effect refers to the simultaneous secretion of two types of EVs with different cargos by the SCs to mediate homeostasis.

#### Stem Cell-Derived Membrane Particles as Drug Delivery Carrier Targeting of Monocytes

Membrane particles (MPs) derived from human adipose MSCs (AD-MSCs) were rarely taken up by lymphocytes, although they could selectively bind to and fuse with plasma membrane of monocytes to specifically induce apoptosis of pro-inflammatory CD14+CD16+ monocytes. However, no such effect was exerted on classical CD14+CD16– monocytes (48) (Table 1). Thus, SC-MPs may act as natural drug delivery vehicles targeting monocytes.

### Microglia

As the resident macrophages of the central nervous system (CNS), microglia play a vital role in regulating inflammation, balancing immunity, and promoting development and tissue repair. It is believed that an M1/M2 phenotype imbalance occurs in the CNS diseases and that the polarization of microglia from the M1 to M2 phenotypes can maintain immune homeostasis and neurological function in patients with CNS diseases (103).

#### Involvement of Neural Stem Cells, Neural Stem Cell-Derived Extracellular Vesicles, and Microglia in Central Nervous System Development

Microglia are the innate immune cells that play an important physiological role in the nervous system (NS). Neural stem cells (NSCs) and neural stem cell-derived extracellular vesicles (NSC-EVs) are closely associated with microglia during neonatal brain development. For example, the EVs released by neonatal sub-ventricular zone (SVZ)-derived NSCs were observed to contain a variety of miRNAs and preferentially induced a transition of CD11b+ microglia to a non-stellate morphology, accompanied by an alteration in the microglial transcriptional state. Conversely, EV-treated neonatal microglia inhibited NSC proliferation by upregulating Let-7-mediated cytokine release (104). Therefore, neonatal NSC-EVs affect the morphology and function of microglia with formation of a negative feedback loop of NSCs that might be conducive to normal development of the NS.

#### Stem Cell-Derived Extracellular Vesicle Regulatory Potential in Immunoreactive Microglia

SC-EVs have been observed to regulate the activation of microglia in a variety of NS disease models (46, 57, 58, 85, 86, 105). For example, MSC-EVs suppressed the activated microglia by inhibiting the phosphorylation of mitogen-activated protein kinase (MAPK) family members extracellular signal kinase 1/2 (ERK1/2), c-Jun N-terminal kinases (JNKs), and the p38 molecules in microglia (46, 57, 85) (Table 1). Notable studies have reported that BM-MSC exosomes could repair spinal cord injury by suppressing the activation of A1 neurotoxic reactive astrocytes induced by activated microglia (86) or by inhibiting the complement system (105) and the NF-κB signaling pathway (46, 57, 105). Meanwhile, SC-EVs have been observed to polarize microglia from classic M1 to anti-inflammatory M2 phenotypes (59, 85, 106, 107), which might be attributed to the targeted suppression of the 3′-UTR mRNA expression in Beclin-1 and Atg5 and inhibition of autophagy-mediated microglial polarization toward pro-inflammatory state by miR-30d-5p-expressing EVs (59) (Table 1). Thus, SC-EVs create a microenvironment conducive to nerve cell repair by inducing expression of microglial immunotolerance phenotypes in NS diseases.

### T Cells

#### The Diversity of Stem Cell-Derived Extracellular Vesicle Immunoregulatory Potential in T Cells

The immunoregulatory effects exerted by SC-EVs on activated T cells remain a widely debated topic. For instance, one study observed that the co-culture of SC-EVs with peripheral blood mononuclear cells (PBMCs) specifically suppressed the proliferation of T cells, whereas it did not affect that of B cells and NK cells (71). However, other studies have reported that SC-EVs inhibited the proliferation of NK cells and B cells, although its effect on the proliferation of T cells remains unclear (30, 108). There are evidences that indicate that SC-EVs do not suppress T cell proliferation; however, they induce upregulation of Tregs and downregulation of pro-inflammatory cytokines (51, 74, 109). Results from most studies clearly indicate that SC-EVs indirectly affect T cells by interaction with macrophages or DCs (110, 111); yet limited number of studies have directly examined the suppressive effects of SC-EVs on T cells (112).

#### Regulatory Potential of Stem Cell-Derived Extracellular Vesicles on T Cells Affected by Different Inflammatory Conditions

Various inflammatory conditions influence the effects of SC-EVs on T cells. For example, exosomes secreted by TGF-β- and IFN-γ-conditioned MSCs significantly inhibited PBMCs and effectively promoted differentiation of T cells into Tregs to alleviate undesirable inflammation, which might be further attributed to upregulation of IL-10, IFN-γ, and IDO in EVs after exposure to TGF-β and IFN-γ (113). In addition, MVs secreted by IFN-γ-conditioned or non-IFN-γ-conditioned human umbilical cord blood MSCs (UCB-MSCs) exerted similar immunosuppressive effects on T cells *in vitro*. However, in mice model with renal ischemic reperfusion, only non-IFN-γ-conditioned MVs attenuated the inflammatory injury *in vivo*. Mass spectrometry revealed that the protein content in IFN-γ-conditioned MVs underwent a significant alteration that might have led to triggering the innate or acquired immune response after inflammatory conditioning. A possible explanation for the above findings could be that the EVs secreted by the same cells under different external conditions originate from different internal vesicle routes (114); for instance, therapeutic MSC-EVs originate from the lipid raft microdomain in the plasma membrane (115).

#### Stem Cell-Derived Extracellular Vesicle Potential in T Cell Proliferation and Activation

##### Inhibition of T cells

It has been demonstrated that SC-EVs carry a variety of active molecules, such as TGF-β (10, 92), active CD73 protein (11, 79), IDO protein (88), or miR-125a-3p (71). These molecules endow SC-EVs with the ability to inhibit T cell proliferation (11, 71, 88, 92, 98, 112, 116, 117) and activation (71, 76, 77, 97, 98, 112, 116), and preserve the circulating naive T cells (71) (Table 1). Studies have revealed that adenosinergic immunosuppression by SC-EVs required co-operation with T cells (11), resulting from the presence of adenosine 5′-triphosphate (ATP) in the extracellular environment during *in vivo* tissue injury. CD73 expressed in EVs has ATPase activity that catalyzes active production of adenosine from adenosine 5′-monophosphate (AMP) (11). Meanwhile, activated T cells expressing CD39 efficiently catalyzed conversion of ATP to AMP (11) (Table 1). Adenosine is highly immunosuppressive. In brief, MSC-EVs suppressed *in vitro* T cell proliferation through adenosinergic signaling (11). Recent studies provide evidence that active molecule TGF-β1 expressed on SC-EV membrane could synergize with adenosine signaling to suppress the proliferation of CD4+ T cells (92) (Table 1). Advances in SCs technology have provided an interesting perspective on the field of transplantation. For instance, in the rat model of myocardial infarction, beta-2 microglobulin (B2M) negative UC-MSC lost the ability to induce CD8+ T cell immune rejection response by the B2M-UCMSC exosomes/miR-24/Bcl-2-like protein 11 (Bim) pathway after B2M-UCMSC injection to the heart (43). The engineered SCs may reveal a novel strategy for tissue repair and regeneration without the requirement for HLA matching. Based on the immunoregulatory effects of SC-EVs on T cells *in vitro* and *in vivo*, these studies have provided guidance for use of SC-EVs therapy in T cell-mediated immune diseases.

##### Promotion of T cells

SC-EVs have been shown to promote the proliferation and activation of T cells. For example, in mice with type 1 diabetes, islet MSC-derived exosomes were observed to activate APCs and autologous T and B cells in islets, increase their respective specific memory cells, and induce IFN-γ production, thus accelerating islet destruction (118). This evidence provides a perspective that certain SC-EVs serve as autoantigen carriers and trigger autoimmune responses. Additionally, The β-catenin-loading (56) or GM-CSF (granulocyte-macrophage colony-stimulating factor)-expressing (90) EVs derived from SCs could promote T cell antitumor response. For example, the exosomes derived from rat AD-MSCs were observed to accelerate intratumoral CD8α+ type I NK-T cell migration and increase circulating NK-T cells to exert antitumor immunity in rats with hepatocellular carcinoma (HCC) (56) (Table 1). In mice with transplanted lung adenocarcinoma, exosomes derived from GM-CSF-expressing embryonic SCs (ESCs) were observed to suppress the migration of immunosuppressive Tregs, whereas they reinforced migration of tumor-reactive CD8+ T effector cells toward intratumor spaces and elevated intratumoral cytokine responses of TNF-α and IFN-γ, contributing to the clearance of foreign components (90) (Table 1). Therefore, EVs derived from engineered SCs may be utilized as a preventive vaccine against the risk of cancer development in human beings.

#### Stem Cell-Derived Extracellular Vesicle Potentials in T Cell Differentiation

##### Stem cell-derived extracellular vesicles regulate Th1/Th2 balance

On the one hand, SC-EVs have been reported to induce the immune response of T helper type 1 (Th1) conversion to T helper type 2 (Th2). For example, SC-EVs drove the shift from Th1 toward Th2 cells and reestablished Th1/Th2 homeostasis by downregulating pro-inflammatory TNF-α and INF-γ and upregulating anti-inflammatory IL-10 or IL-4 (29, 44, 51, 74, 76) (Table 1). Moreover, SC-EVs could also regulate Th2 immune response toward Th1. For example, in the early stage of allergic asthma, the immune response mediated by Th2 cells was primarily through eosinophilic infiltration (49). Human AD-MSC-EVs were observed to downregulate eosinophil infiltration and IL-4, IL-5, and TGF-β levels, whereas they did not affect IFN-γ and IL-10 in the bronchoalveolar lavage fluid (BALF) (49). The advanced acute severe refractory asthma is a mixed immune response by Th2/Th17, and an allergic airway inflammation mediated by neutrophils and eosinophils. Human BM-MSC-EVs were observed to inhibit the infiltration of neutrophils and eosinophils and downregulate IL-4, IL-5, and IL-17 expression while upregulating IFN-γ and IL-10 expression in BALF (75). The possible mechanism is that SC-EVs shift the inflammatory responses from Th2 or Th2/Th17 toward upregulation of counter-regulatory Th1 response and/or secretion of anti-inflammatory mediators, such as IL-10 (49, 75) (Table 1). Based on the immune balance effect exerted by SC-EVs on Th1/Th2 cells, these studies provide the basis for preclinical trials of Th1/Th2 immune response disorders.

##### Stem cell-derived extracellular vesicles regulate Th17/Treg balance

SC-EVs regulate Th17/Treg balance such as inhibition the differentiation of activated CD4+ T cells into Th17 cells, downregulation pro-inflammatory IL-17, promotion differentiation of Tregs (31, 51, 74, 76, 94) (Table 1) and CTLA-4+ Tregs (74), upregulation anti-inflammatory TGF-β, and inhibition aberrant inflammatory responses in stroke (31), type 1 diabetes (51, 76), and chronic periodontitis (94). A possible underlying mechanism could be the immune equilibrium controlled by PGE2 and TGF-β (76) signaling pathways or miRNA-155-5p (94) in EVs. A second underlying mechanism could be the induction of phenotypic transition of macrophages into M2 to regulate T cells indirectly by EVs derived from human NSCs (31) (Table 1). Considered in conjunction, SC-EVs alleviate the inflammatory microenvironment through Th17/Treg regulatory network. PGE2 protein, TGF-β protein, and miR-155-5p may act as promising therapeutic targets against immune imbalance.

##### Stem cell-derived extracellular vesicles inhibit naive T cell differentiation into Th1, Th2, Th17, Tfh, and Tc1 cells and upregulate Tregs

In type 1 diabetes, SC-EVs were reported to downregulate IFN-γ and IL-17 (77, 97), upregulate IL-10 and TGF-β (77), inhibit activated T cell differentiation into Th1 and Th17 cells (77, 97) (Table 1), and increase Treg expression (77), thereby inducing immunotolerance. Additionally, in the Sjögren syndrome disease model, SC-EVs were observed to prevent disease progression by repressing differentiation of naive CD4+ T cell into T follicular helper (Tfh) and Th17 cells (99) (Table 1). In a contact hypersensitivity (CHS) mouse model, hUC-MSC-EVs were observed to inhibit CD8+IFN-γ+ cytotoxic T (Tc1) cells and Th1 cell immune responses and to induce Treg expression (45). One underlying mechanism could be that SC-EVs inhibit T cell differentiation into Th1 cells (98) or Th2 cells (52) (Table 1) while promoting Treg differentiation (91) (Table 1) by inducing phenotypic transformation of APCs (52, 77, 91, 97–99). Another underlying mechanism could be that SC-EVs regulate the expression of the related genes involved in inflammation and immune cell development; for example, they could upregulate miR-let-7b and miR-let-7d and downregulate miR-155 in Treg cells (119). Additionally, the study on MSC transplantation (MSCT) in tight-skin mice model demonstrated that BM-MSCs in recipients could take up and reuse miR-151-5p loaded in the MSC-EVs of donors to inhibit IL4Rα/mTOR pathway, downregulate IL-4, inhibit Th2 cell differentiation and infiltration, and contribute to the rebuilding of BM-MSC function and BM homeostasis (78) (Table 1). These findings delineate EV-mediated immune responses for cross talk between SC-T cell subsets that provide potential therapeutic targets for autoimmune diseases.

#### Stem Cell-Derived Extracellular Vesicle Potential in T Cell Apoptosis

While SC-EVs were observed to exert no effect on T cell proliferation, they induce T cell apoptosis (74, 79, 109), possibly through an SC-EV-mediated mechanism *via* adenosine A2A receptor (79) (Table 1). For example, in a study on human-into-mouse xenogeneic chronic and severe GVHD model, a significant increase was observed in pathogenic CD39+ Th1 cell population. Human BM-MSC-derived CD73+ exosomes were observed to function synergistically with CD39-expressing Th1 cells to accelerate massive accumulation of adenosine signals, resulting in specific apoptosis of adenosine A2A receptor-expressing Th1 cells, thereby downregulating IFN-γ and TNF-α. This resulted in the attenuation of inflammatory response *in vivo* (79) (Table 1). Collectively, the findings propose a significant EV-mediated cross talk between SCs and T cells by purinergenic signaling, which sheds light on the potential of EV-based therapeutic approach against immunological diseases.

#### Stem Cell-Derived Extracellular Vesicle Potential in T Cell Migration and Infiltration

Despite the limited effect exerted on the proliferation and activation of autologous T cells, SC-EVs have been shown to inhibit the infiltration of T cells in lesions (42, 54), thus attenuating inflammatory injury. This could be attributed to the downregulation of C-C motif chemokine ligand-21 (CCL-21, capable of attracting T cells) expression mediated by human UC-MSC exosomes (42) (Table 1). This could also be attributed to the inhibitory effect exerted by AD-SC-EVs on the adhesion and trafficking of pathogenic T cells in spinal cord pial venules in early stages of the disease through interference with the integrin-dependent chemokine-induced signal transduction pathways without affecting adhesive molecule expression (54) (Table 1).

### B Cells

#### The Diverse Immunoregulatory Effects of Stem Cell-Derived Extracellular Vesicles on B Cells

In a co-culture of activated PBMCs and MSC-EVs, preferential uptake of EVs by B cells exerted a stronger inhibitory effect on the proliferation of B cells than on other immune cells (30). In a similar experiment, MSC-EVs were internalized only by activated CD19+/CD86+ B cells to inhibit proliferation, differentiation, and antibody production of B cells and to hinder memory B cell maturation (120). However, under normal or hypoxic conditions, human amniotic fluid SC-derived EVs (AFSC-EVs) exerted limited inhibitory effect on proliferation of activated PBMCs, whereas they exerted significant immunoregulatory effects by inhibiting maturation of CD27+CD19+ memory B cells (121).

#### Stem Cell-Derived Extracellular Vesicle Potential in B Cell/Plasma Cell Proliferation and Activation

SC-EVs were observed to suppress B cell proliferation (30, 80, 81, 108, 120), activation (81, 83) and migration (81) in order to induce anti-inflammatory immune responses. *In vitro*, SC-EVs have been observed to exert immunosuppressive effects by mediating differential mRNA expression of relevant genes in activated B cells (80) (Table 1) or by downregulating PI3K-AKT signaling pathway and inhibiting actin activation in B cells *via* the delivery of miR-155-5p (81) (Table 1). Additionally, SC-EVs could also interact with tumor B cells [in chronic lymphocytic leukemia (CLL)]. For instance, BM-MSC-EVs were reported to induce CLL B cell gene expression profile modification, promote CLL B cell survival and their migration, and rescue them from apoptosis (82) (Table 1). The promotive effect of SC-EVs on CCL B cells serves as a basis for the exploration of a therapeutic target for hematological diseases. The study (82) that mechanistically links CLL B cells and SC-EVs with disease progression has provided a pathophysiologically relevant context or the acquired immunomodulatory activity of SCs.

#### Stem Cell-Derived Extracellular Vesicle Potentials in B Cell/Plasma Cell Differentiation

SC-EVs were observed to inhibit B cell/plasma cell (PC) differentiation and antibody production (29, 80, 83, 108) and to induce production of IL-10-expressing regulatory B cells (Bregs) (29, 83), thus inducing anti-inflammatory immune response. These findings are suggestive of the ability of SCs to suppress B cell inflammation. In addition, human primary BM-MSCs mediate *in vitro* differentiation and maturation of circulating antibody-secreting cells (ASCs) to BM long-lived PCs (LLPCs), possibly through MSC-EVs (84), thereby promoting the survival of ASC or PCs in peripheral blood collected from healthy subjects and facilitating IgG secretion (3, 84) (Table 1). This might be attributed to the utilization of EV-mediated delivery of signaling proteins (fibronectin-1, YWHAZ), a proliferation-inducing ligand (APRIL), and hypoxic conditions in the peripheral environment by post-irradiated MSC to facilitate LLPC survival by downregulation of mTORC1 signaling and upregulation of hypoxia signatures (84). Understanding the mechanisms of human PC differentiation and maintenance will facilitate *in vitro* culture of antibodies in the near future.

## Concluding Remarks and Future Perspectives

As discussed in this review, the immunoregulatory potential of SC-EVs against immune cells is dependent on cell type, cellular status, the maturity of origin cells, and the type of EVs, among other factors. However, in multiple studies, SC-EVs were reported to inhibit immune cell production and promote an immunotolerant microenvironment. The immune responses regulated by SC-EVs are comparable with those mediated by stem/progenitor cells. Treatment using EVs has multiple advantages over cell therapy, like their small size, which prevents entrapment in filter organs like the lungs, liver, and spleen. Moreover, the membrane-binding property of EVs imparts exceptional biocompatibility and biostability to the encapsulated cargos. As a promising candidate for novel cell-free therapy, EVs may be widely used as an alternative to SCs in management of a variety of immunity-related diseases for maintenance of the microenvironment for tissue homeostasis and tissue regeneration upon injury. However, there are multiple questions that remain unanswered. For example, how do SC-EVs home damaged tissues? Or how do SC-EV-transferred specific miRNAs target the genes in recipient cells? So far, different animal models have been used in multiple studies to investigate the immunoregulatory roles of stem/progenitor cell-derived EVs; yet limited clinical studies have been conducted on application of SC-EVs to human subjects (122). Therefore, an extensive body of research is necessary before we adopt large-scale application of SC-EVs in clinical practice.

## Author Contributions

MX and ZS prepared the table and figure. MX, WX, ZW, AA, WW, and CW drafted the manuscript. MX, WX, and CW edited and revised the manuscript. All authors contributed to manuscript revision and read and approved the submitted version.

### Conflict of Interest

The authors declare that the research was conducted in the absence of any commercial or financial relationships that could be construed as a potential conflict of interest.
